# Study on the mechanism of LOXL1-AS1/miR-3614-5p/YY1 signal axis in the malignant phenotype regulation of hepatocellular carcinoma

**DOI:** 10.1186/s13062-021-00312-8

**Published:** 2021-12-04

**Authors:** ZhenYu Feng, ZhenYu Ye, JiaMing Xie, Wei Chen, Wei Li, ChunGen Xing

**Affiliations:** 1 Department of Hepatobiliary Surgery, The Second Affiliated Hospital of Soochow University, Suzhou, 215004 Jiangsu China; 2 Department of General Surgery, The Second Affiliated Hospital of Soochow University, No. 1055 Sanxiang Road, Gusu District, Suzhou, 215004 Jiangsu China

**Keywords:** Hepatocellular carcinoma, LOXL1-AS1, miR-3614-5p, YY1

## Abstract

**Background:**

Hepatocellular carcinoma (HCC) is one of the most common malignant tumors with high mortality worldwide. Accumulating researches have indicated that long non‑coding RNAs (lncRNAs) are involved in varies human cancers, including HCC. Nevertheless, the specific molecular mechanism of lncRNA lysyl oxidase like 1 antisense RNA 1 (LOXL1-AS1) in HCC is still unclear.

**Methods:**

LOXL1-AS1 expression was tested via qRT-PCR in HCC cells. Functional and mechanism assays were respectively done to evaluate the biological functions of HCC cells and the potential interaction of LOXL1-AS1 and other factors.

**Results:**

We discovered that LOXL1-AS1 was high expressed in HCC cells. Inhibition of LOXL1-AS1 repressed cell proliferation, migration and invasion, but enhanced cell apoptosis in HCC. Further, miR-3614-5p was proven to be sponged by LOXL1-AS1. Additionally, Yin Yang 1 (YY1) was proven as the target gene of miR-3614-5p, and YY1 depletion could repress HCC cell malignant behaviors. YY1 could also transcriptionally activate LOXL1-AS1 expression. In rescue assays, we confirmed that overexpression of YY1 or miR-3614-5p inhibition could reverse the suppressive effects of LOXL1-AS1 silence on the malignant behaviors of HCC cells.

**Conclusion:**

In short, LOXL1-AS1/miR-3614-5p/YY1 forms a positive loop in modulating HCC cell malignant behaviors.

**Supplementary Information:**

The online version contains supplementary material available at 10.1186/s13062-021-00312-8.

## Background

Hepatocellular carcinoma (HCC) is one of the most common malignant tumors [1]. It has gradually become the second leading cause of cancer-related deaths in the whole world [2]. With the development of treatment methods and the improvement of screening technology, morbidity and mortality of HCC have been controlled to a certain extent [3, 4]. However, the risk of postoperative recurrence and tumor metastasis keeps the 5-year survival rate at a low level [5]. At present, the explicit HCC pathogenesis has not been clarified [6]. Thus, it is necessary to explore the molecular mechanism of HCC in depth and discover more effective targets for treatment, so as to improve the survival rate of patients.

Long non-coding RNAs (lncRNAs) are defined as a group of RNA with length over 200 nucleotides which have no ability of coding protein [7]. They are the vital factors in the regulation of gene expression by means of epigenetic modification, transcriptional regulation and post-transcriptional regulation [8]. Mounting studies have unveiled that lncRNAs are involved in assorted cell processes and the dysregulation of lncRNAs is closely connected to cancer biology activities [9–11]. For example, upregulation of lncRNA HOTAIR can accelerate cell migration and invasion of breast cancer by the miR-20a-5p/HMGA2 axis [12]. LncRNA TUC338 has been proved to facilitate cell invasion in lung cancer through activating MAPK pathway [13]. Further, it has also been indicated that lncRNA CDKN2BAS predicts the unfavorable prognosis of HCC patients and expedites cell metastasis through the miR-153-5p/ARHGAP18 axis [14]. Similarly, lncRNA lysyl oxidase like 1 antisense RNA 1 (LOXL1-AS1) has been frequently studied in varies cancer types and has also been confirmed to play carcinogenic effect in some cancers. For instance, LOXL1-AS1/miR-28-5p/SEMA7A axis has been discovered to accelerate cell growth of pancreatic cancer [15]. LOXL1-AS1 has been reported to expedite cell stemness in gastric carcinoma through regulating miR-708-5p/USF1 axis [16]. Also, LOXL1-AS1 has been validated to promote cell proliferation in non-small cell lung cancer via the regulation of miR-3128/RHOXF2 axis [17]. Importantly, recent study has illustrated that LOXL1-AS1 can accelerate cell proliferation and invasion in HCC [17], while its specific regulatory mechanism in the modulation of HCC cell malignant behaviors has not been clarified.

MicroRNAs (miRNAs) are a class of noncoding RNAs consisting of 18–25 nucleotides. They can negatively modulate the expression and translation of target messenger RNAs (mRNAs) [18, 19]. Dysregulation of miRNAs is associated with assorted cancer development. For instance, miR-218 has been confirmed to repress EMT process of HCC cells through targeting SERBP1 [20]. However, miR-5692a has been verified to promote HCC cell proliferation and repress cell apoptosis via targeting HOXD8 [21]. MiRNAs can also interact with lncRNAs to take part in the regulation of different cancers. For example, it has been reported that lncRNA FAL1 accelerates cell proliferation and migration via acting as a sponge of miR-1236 in HCC [22]. Therefore, whether LOXL1-AS1 might function as a sponge of certain miRNA to exert its specific influences on the biological functions of HCC cells remains to be explored.

In this study, we aimed to investigate the regulatory role of LOXL1-AS1 in HCC cells. Further, the interaction of LOXL1-AS1 and miRNAs was also deeply explored.

## Results

### LOXL1-AS1 accelerates cell proliferation, migration and invasion but represses cell apoptosis in HCC

**Fig. 1:**
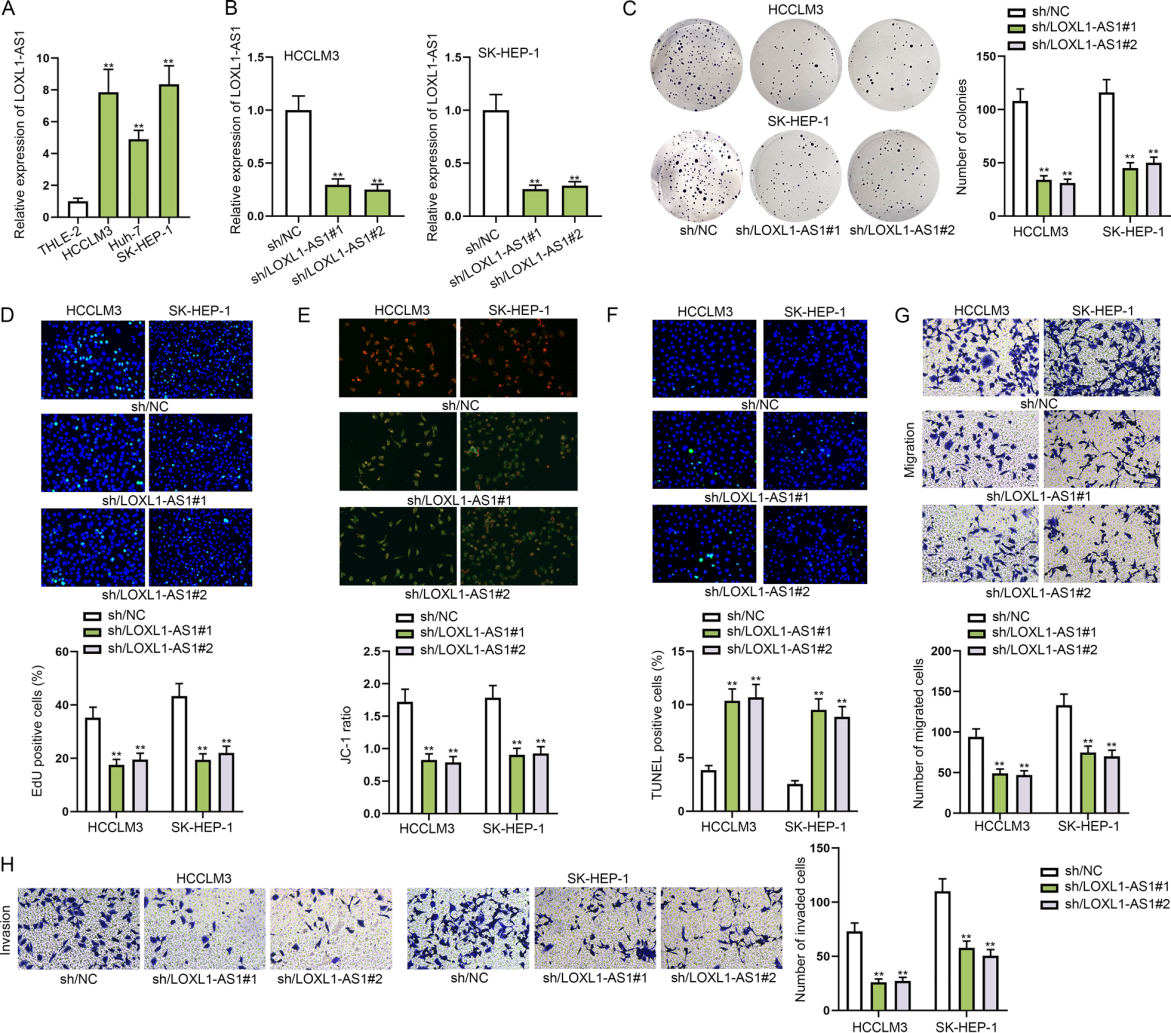
LOXL1-AS1 accelerates cell proliferation, migration and invasion but represses cell apoptosis in HCC. **A** The expression level of LOXL1-AS1 in HCC cell lines and normal cell line was tested by qRT-PCR. **B** The interference efficiency of sh-LOXL1-AS1 in HCCLM3 and SK-HEP-1 cells was detected via qRT-PCR. **C** Colony formation assay estimated cell proliferative capacity in response to transfection of sh/LOXL1-AS1 and sh/NC. **D** EdU assay measured cell proliferation before and after silencing LOXL1-AS1. **E**, **F** JC-1 assay and TUNEL assay were utilized to measure apoptosis of cells transfected with sh/LOXL1-AS1 and sh/NC. **G**, **H** Transwell assay detected cell migratory and invasive capacities when LOXL1-AS1 was knocked down. **P < 0.01

LOXL1-AS1 has been identified to be dysregulated in assorted cancers and exert the regulatory function on cancer progression. Data obtained from ENCORI (http://starbase.sysu.edu.cn/index.php) reflected aberrantly higher expression of LOXL1-AS1 in liver hepatocellular carcinoma (LIHC) tissues compared with normal tissues (*p* = 0.0099, Additional file 1: Fig. S1A). Thereafter, we detected LOXL1-AS1 expression in HCC cell lines (HCCLM3, Huh-7 and SK-HEP-1) and normal liver cell line (THLE-2) through qRT-PCR analysis. As a result, LOXL1-AS1 was high expressed in HCC cell lines in comparison with THLE-2 (Fig. 1A). LOXL1-AS1 showed higher expression in HCCLM3 and SK-HEP-1 cells, and thus we selected the two cell lines for the following assays. We knocked down LOXL1-AS1 in HCCLM3 and SK-HEP-1 cells by transfecting specific shRNAs targeting LOXL1-AS1 (sh/LOXL1-AS1#1/2) and the results of qRT-PCR suggested that LOXL1-AS1 expression was obviously decreased after sh/LOXL1-AS1 transfection (Fig. 1B). Subsequently, a series of functional assays were performed to evaluate the effects of LOXL1-AS1 knockdown on HCC cell proliferation, apoptosis, migration and invasion. Colony formation assay illustrated that the quantity of colonies obviously declined as a result of transfection with sh/LOXL1-AS1, which indicated that cell proliferation was repressed by LOXL1-AS1 depletion (Fig. 1C). Then EdU assay further proved the repressive influences exerted by sh/LOXL1-AS1 on cell proliferative capability since we observed that EdU positive cells were significantly reduced in sh/LOXL1-AS1 transfection groups (Fig. 1D). Next, as the decline of mitochondrial membrane potential is the sign of early period of cell apoptosis [23], JC-1 assay was performed in order to detect cell apoptosis, and we discovered that the ratio of red/green fluorescence was notably reduced by LOXL1-AS1 down-regulation, suggesting cell apoptosis was enhanced by LOXL1-AS1 knockdown (Fig. 1E). Further, it was manifested from TUNEL assay that the percentage of TUNEL positive cells was increased under the influence of LOXL1-AS1 silence, further proving LOXL1-AS1 deficiency led to strengthened cell apoptosis (Fig. 1F). Flow cytometry was also performed for directly reflecting influence of LOXL1-AS1 knockdown on cell apoptosis, the result of which indicated enhanced cell apoptosis after sh/LOXL1-AS1 transfection (Additional file 1: Fig. S1B). Moreover, transwell assays were employed for estimating cell migration and invasion capability. The results indicated that the transfection of sh/LOXL1-AS1 obviously reduced the quantity of migrated cells and invaded cells, suggesting cell migratory and invasive capabilities was suppressed by LOXL1-AS1 depletion (Fig. 1G, H). Thus, we proved the upregulation of LOXL1-AS1 in HCC and upregulated LOXL1-AS1 could accelerate cell proliferation, migration and invasion, while inhibiting cell apoptosis.

### LOXL1-AS1 acts as a ceRNA to sponge miR-3614-5p in HCC cells

**Fig. 2:**
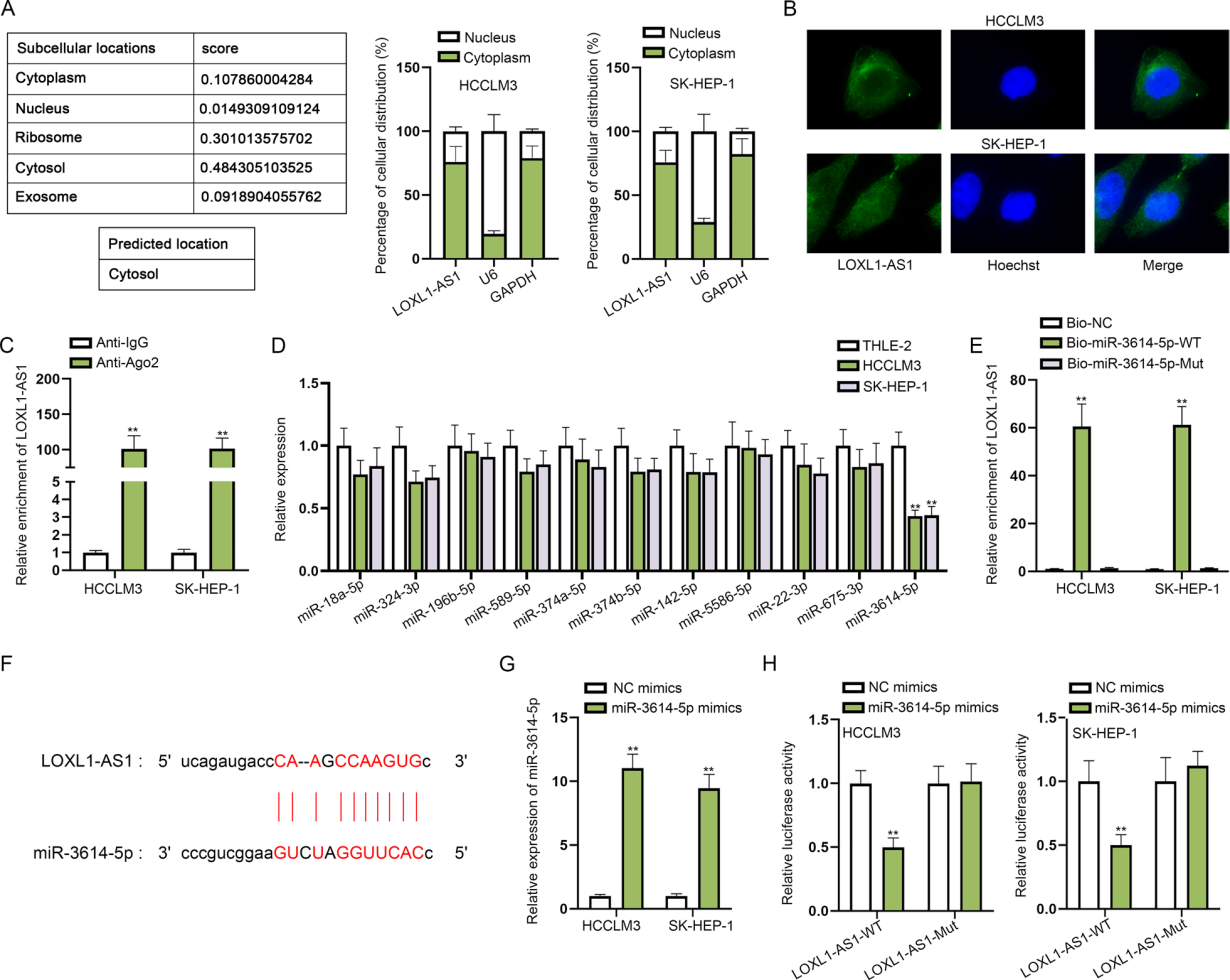
LOXL1-AS1 acts as a ceRNA to sponge miR-3614-5p in HCC cells. **A**, **B** Subcellular fractionation assay and FISH assay, supported with bioinformatics analysis (LncLocator), were done to determine the distribution of LOXL1-AS1 in HCC cells. **C** In RIP assay, the enrichment of LOXL1-AS1 in Ago2 group was measured by qRT-PCR. **D** qRT-PCR analysis tested the expression of candidate miRNAs in THLE-2, HCCLM3 and SK-HEP-1 cells. **E** RNA pull down assay was utilized to verify the combination of LOXL1-AS1 and miR-3614-5p. **F** The binding sites of miR-3614-5p and LOXL1-AS1 were obtained from ENCORI database. **G** The overexpression efficiency of miR-3614-5p mimics was detected by qRT-PCR. **H** Luciferase reporter assay validated the binding of miR-3614-5p and LOXL1-AS1 in HCC cells. **P < 0.01

In order to determine the regulatory mechanism of LOXL1-AS1 in HCC cells, we first detected the subcellular localization of LOXL1-AS1. Through the prediction of lncLocator (http://www.csbio.sjtu.edu.cn/bioinf/lncLocator/) database, LOXL1-AS1 was predicted to mainly exist in cell cytosol, and the results of subcellular fractionation assay verified the main distribution of LOXL1-AS1 in the cytoplasm of HCCLM3 and SK-HEP-1 cells (Fig. 2A), which was further proved by FISH assay (Fig. 2B). The results mirrored that LOXL1-AS1 might participate in post-transcriptional regulation. Recently, competing endogenous RNA (ceRNA) network has been identified in assorted cancers and play the crucial regulatory function in tumorigenesis and development [24]. Importantly, it is confirmed that lncRNAs can function as a ceRNA to sponge miRNA and regulate mRNA expression at post-transcriptional level [25]. Thus, we suspected that LOXL1-AS1 could act as a ceRNA in HCC cells. The results of RIP assay indicated that LOXL1-AS1 was abundantly precipitated in the anti-Ago2 groups, which preliminarily validated our assumption (Fig. 2C). Next, we utilized ENCORI to predict miRNAs that could bind with LOXL1-AS1 under specific condition (pan-Cancer ≥ 6) and eleven candidate miRNAs were screened out. For further screening, qRT-PCR analyzed the expressions of candidate miRNAs in HCC cells (HCCLM3 and SK-HEP-1) and THLE-2 cells. The results indicated that only miR-3614-5p displayed significantly lower expression in HCCLM3 and SK-HEP-1 cells compared with normal cell line (THLE-2) (Fig. 2D). Moreover, from ENCORI, miR-3614-5p expression was discovered to be apparently lower in LIHC tissues compared with normal tissues (1.2e−11, Additional file 1: Fig. S1C). Accordingly, we selected miR-3614-5p for the later assays. It was illustrated by RNA pull down assay that LOXL1-AS1 was abundantly precipitated in the pulldown of biotinylated miR-3614-5p-WT probe, suggesting LOXL1-AS1 bound to miR-3614-5p in HCC cells (Fig. 2E). Further, we obtained the binding sites of LOXL1-AS1 and miR-3614-5p from ENCORI database (Fig. 2F). Subsequently, we overexpressed miR-3614-5p in HCCLM3 and SK-HEP-1 cells by transfecting miR-3614-5p mimics and qRT-PCR results indicated that miR-3614-5p expression was significantly upregulated after the indicated transection (Fig. 2G). Then luciferase reporter assay manifested that the luciferase activity of LOXL1-AS1-WT could be repressed by miR-3614-5p upregulation, while that of LOXL1-AS1-Mut was almost unchanged under the same condition (Fig. 2H). Overall, these results proved that LOXL1-AS1 directly bound to miR-3614-5p in HCC cells.

### YY1 is the target gene of miR-3614-5p in HCC cells

**Fig. 3:**
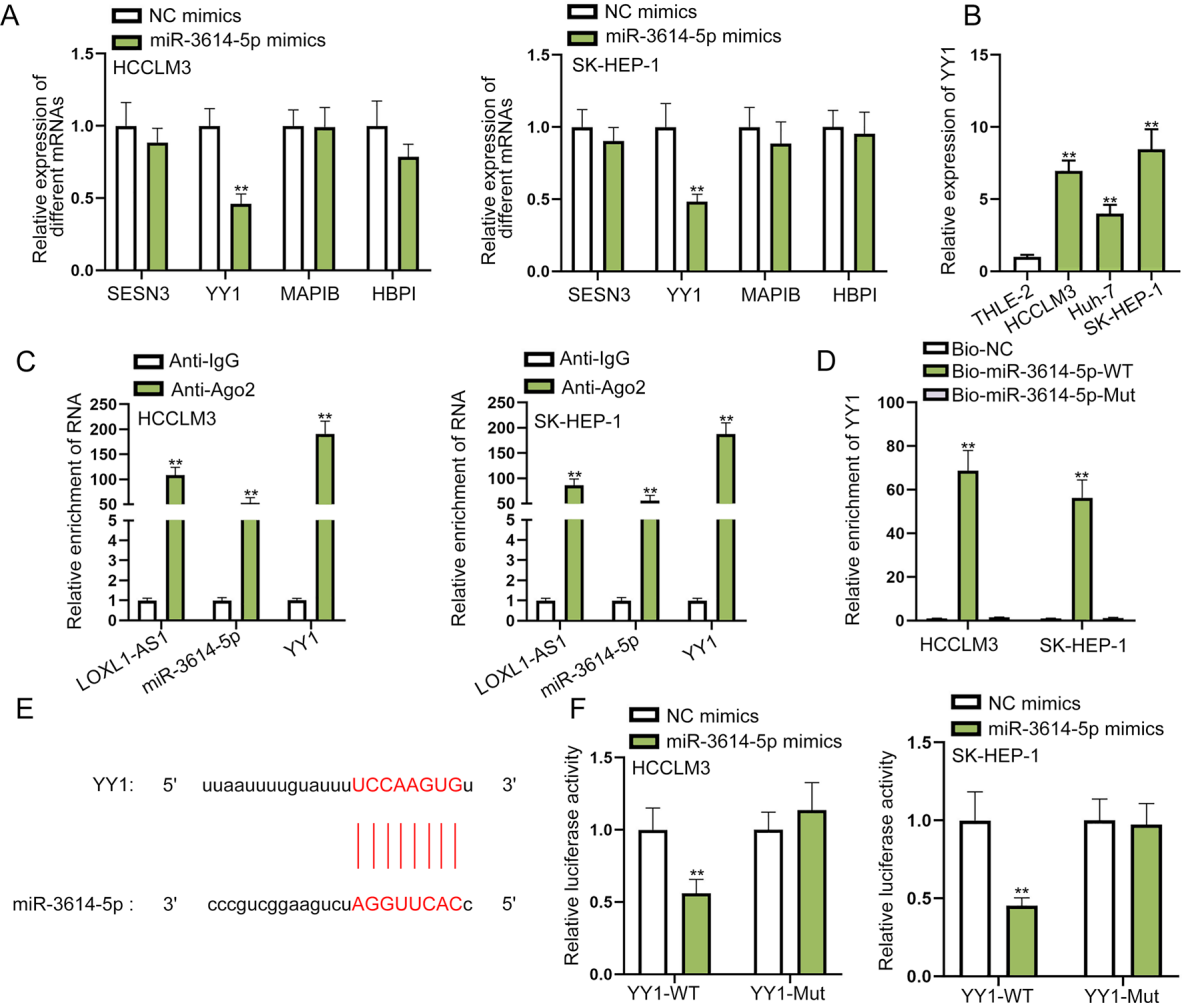
YY1 is the target gene of miR-3614-5p in HCC cells. **A** The expression of candidate mRNAs was tested through qRT-PCR in miR-3614-5p mimics transfected cells. **B** The expression of YY1 in HCC cell lines and normal cell line was detected via qRT-PCR. **C** RIP assay verified the existence of LOXL1-AS1/miR-3614-5p/YY1 in RISC complex. **D** RNA pull down assay further proved the interaction of miR-3614-5p and YY1. **E** The binding sites of miR-3614-5p and YY1 were demonstrated. **F** Luciferase reporter assay proved the combination of miR-3614-5p and YY1 by measuring the luciferase activity of YY1-WT/Mut under the influence of miR-3614-5p mimics transfection. **P < 0.01

We further investigated the downstream mRNA of miR-3614-5p. We utilized ENCORI database to predict the possible mRNAs that may bind with miR-3614-5p. After taking the intersection of microT and miRmap databases, we selected the top four mRNAs of AgoExpNum from all the mRNAs predicted under the indicated condition (pan-Cancer ≥ 10). As a result, SESN3, YY1, MAPIB and HBPI were selected out. For further screening, we detected the expression levels of the four candidate mRNAs in cells transfected with miR-3614-5p mimics. The results of qRT-PCR indicated that only the expression of Yin Yang 1 (YY1) was significantly lessened by miR-3614-5p overexpression, suggesting the negative correlation between miR-3614-5p and YY1 (Fig. 3A). Therefore, YY1 was chosen as our research target. Then, the data of ENCORI indicated that YY1 expression was predicted to be significantly higher in LIHC tissues than in normal tissues (3.5e−16, Additional file 1: Fig. S1D). We also detected YY1 expression in HCC cell lines and normal cell line via qRT-PCR and western blot. As expected, YY1 displayed higher expression in HCC cells compared with THLE-2 cells (Fig. 3B and Additional file 1: Fig. S1E). Then RIP assay was conducted to evaluate the ceRNA regulatory axis in HCC cells, and the results indicated that LOXL1-AS1, miR-3614-5p and YY1 were abundantly precipitated in anti-Ago2 groups, implying the co-existence of LOXL1-AS1/miR-3614-5p/YY1 in RISC complex (Fig. 3C). Further, RNA pull down assay manifested that YY1 could be pulled down by biotinylated miR-3614-5p-WT probe in HCC cells (Fig. 3D). Next, we predicted the binding sites of miR-3614-5p and YY1 from ENCORI (Fig. 3E). Then we observed from luciferase reporter assay that overexpression of miR-3614-5p significantly repressed the luciferase activity of YY1-WT, which further confirmed the combination of miR-3614-5p and YY1 (Fig. 3F). In short, we proved that YY1 was targeted by miR-3614-5p in HCC cells.

### YY1 accelerates cell proliferation, migration, invasion but inhibits cell apoptosis in HCC

**Fig. 4:**
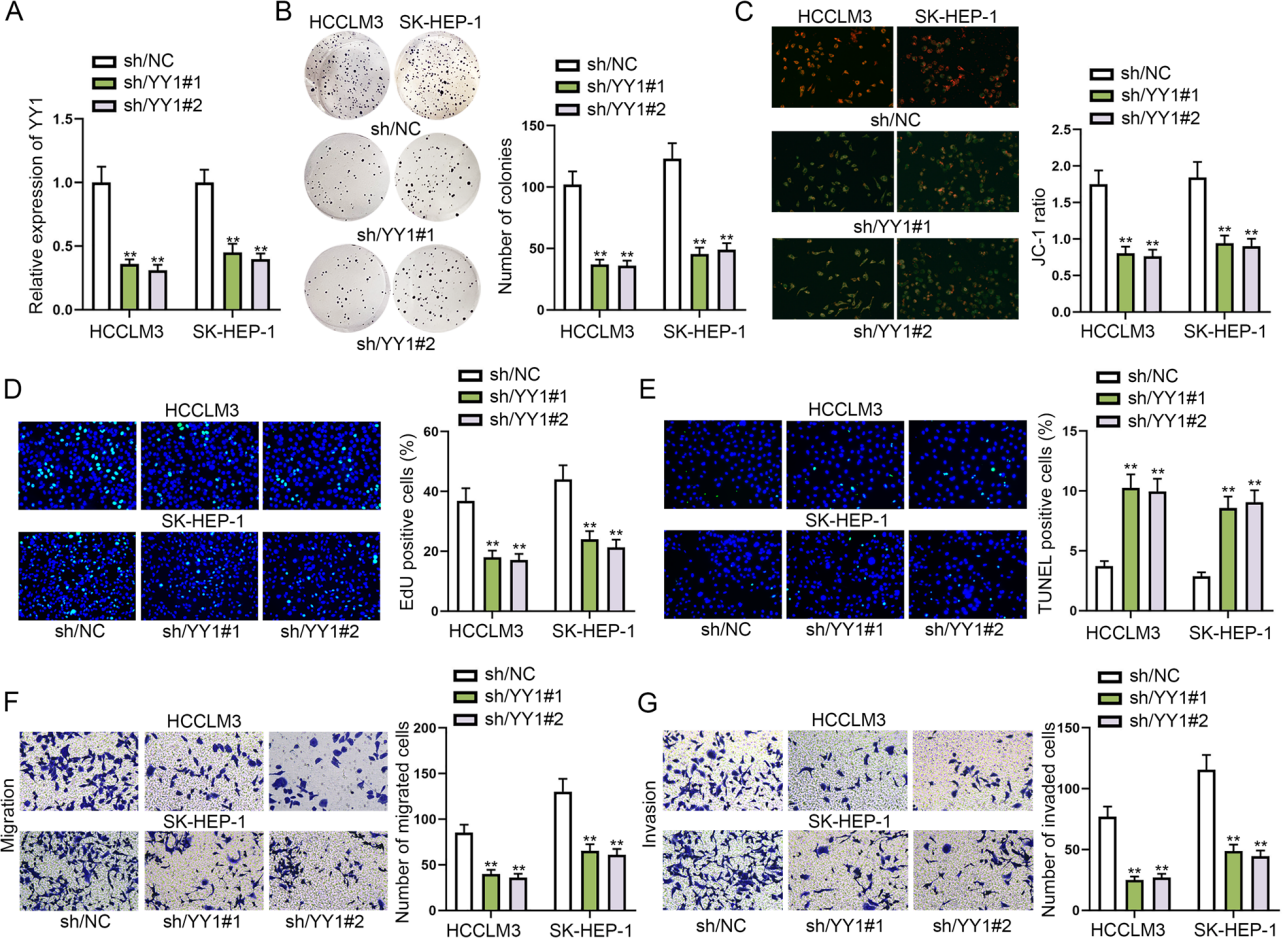
YY1 accelerates cell proliferation, migration, invasion but inhibits cell apoptosis in HCC. **A** YY1 expression in HCC cells transfected with sh/YY1 was tested via qRT-PCR. **B** Cell proliferation was measured by colony formation assay under the influence of YY1 depletion. **C** JC-1 assay was utilized to for the detection of mitochondrial membrane potential when YY1 was silenced in cells to reflect the changes of cell apoptosis. **D** EdU assay estimated the proliferative capacity of sh/YY1-transfetced cells and sh/NC-transfected cells. **E** TUNEL assay detected cell apoptosis after we silenced YY1 in cells. **F**, **G** Transwell assay estimated cell migration and invasion when YY1 was knocked down in cells. **P < 0.01

For the sake of determining the biological function of YY1 in HCC cells, we performed loss-of-functional assays. First, we silenced YY1 in HCCLM3 and SK-HEP-1 cells by transfecting sh/YY1, and qRT-PCR and western blot showed that YY1 expression was obviously decreased after the indicated transfection (Fig. 4A and Additional file 1: Fig. S1F). It was observed in colony formation assay that YY1 knockdown led to a notable decline in the number of colonies, suggesting cell proliferation could be restrained by silencing YY1 (Fig. 4B). Next, JC-1 assay showed that the ratio of red/green fluorescence in cells was reduced by YY1 down-regulation, which implied that cell apoptosis was accelerated by YY1 depletion (Fig. 4C). Additionally, in EdU assay, a decrease in EdU positive cells was discovered as a result of sh/YY1 transfection (Fig. 4D). The results of TUNEL and flow cytometry assays further validated that YY1 deficiency could overtly strengthen the apoptotic ability of HCC cells (Fig. 4E and Additional file 1: Fig. S1G). These results together confirmed that YY1 knockdown repressed cell proliferation but accelerated cell apoptosis. Next, transwell assay indicated that cell migratory and invasive capacities were repressed by YY1 inhibition (Fig. 4F, G). In a word, YY1 accelerated cell proliferation, migration, invasion but inhibited cell apoptosis in HCC.

### LOXL1-AS1 exacerbates HCC cell malignant behaviors via sponging miR-3614-5p to upregulate YY1

**Fig. 5:**
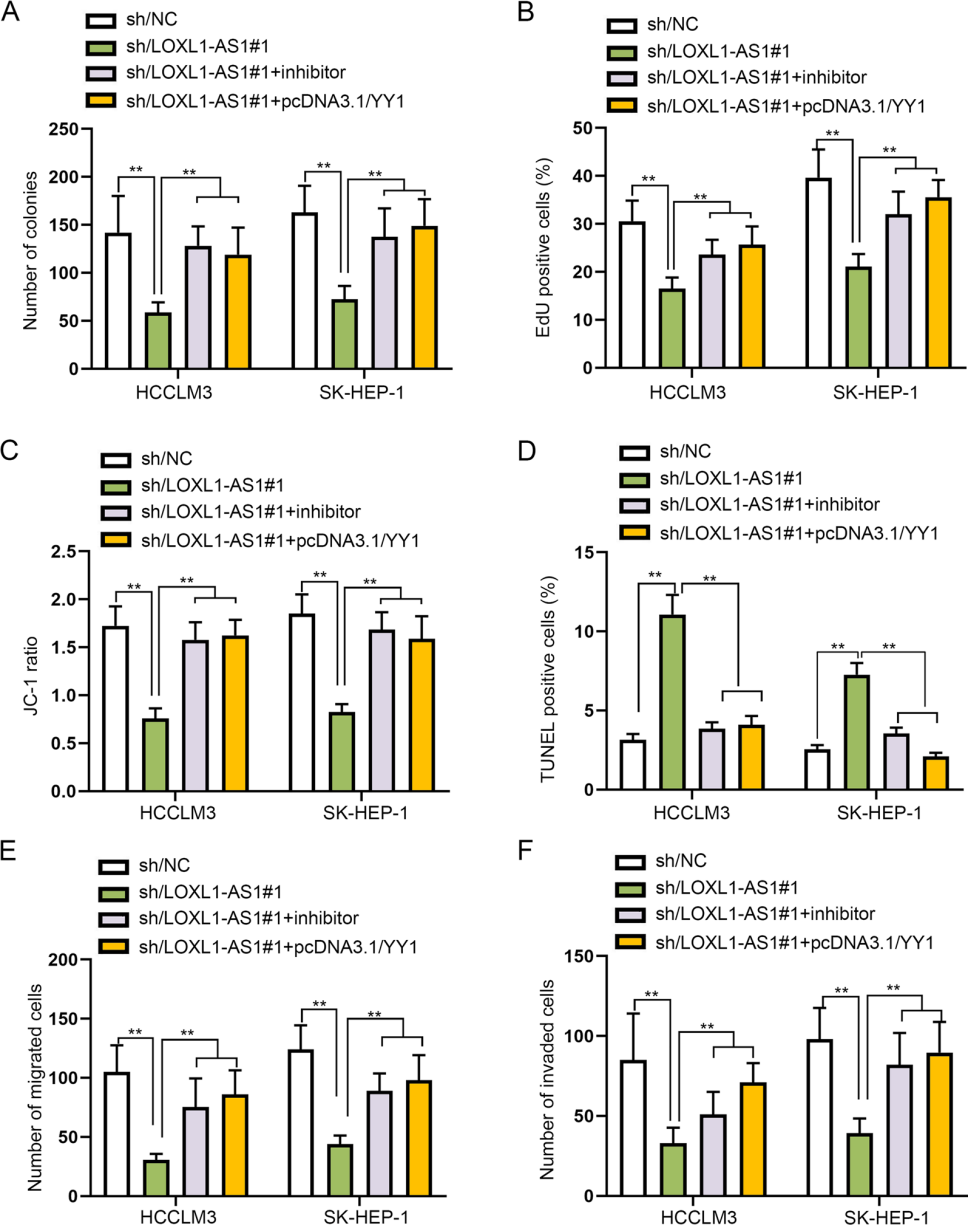
LOXL1-AS1 exacerbates the malignant behaviors of HCC cells via miR-3614-5p/YY1. **A**, **B** Colony formation assay and EdU assay measured cell proliferation in different groups, specifically, sh/NC, sh/LOXL1-AS1#1, sh/LOXL1-AS1#1 + inhibitor and sh/LOXL1-AS1#1 + pcDNA3.1/YY1. **C**, **D** In addition to detection of mitochondrial membrane potential by JC-1 to reflect cell apoptosis, the apoptotic ability of cells was also evaluated via TUNEL assay in different groups. **E**, **F** Transwell assays estimated cell migration and invasion in different groups. **P < 0.01

Subsequently, we carried out rescue experiments to confirm the validity of the regulatory axis of LOXL1-AS1/miR-3614-5p/YY1 in HCC cells. YY1 expression was tested via western blot after cells were transfected with different plasmids, which indicated that YY1 expression mitigated by LOXL1-AS1 knockdown could be rescued after miR-3614-5p inhibition or YY1 augment (Additional file 1: Fig. S1H). After that, colony formation and EdU assays were performed to estimate on cell proliferation under the indicated conditions. According to the colony formation and EdU assay results, we discovered that cell proliferation repressed by LOXL1-AS1 depletion could be totally reversed by miR-3614-5p inhibition or YY1 overexpression (Fig. 5A, B and Additional file 2: Fig. S2A-B). Then, it was illustrated from JC-1, TUNEL and flow cytometry assays that cell apoptosis enhanced by LOXL1-AS1 down-regulation was fully reversed by co-transfection of miR-3614-5p inhibitor or pcDNA3.1-YY1 (Fig. 5C, D and Additional file 2: Fig. S2C-E). Furthermore, transwell assay proved that miR-3614-5p repression or YY1 upregulation could fully counteract the inhibitory effect of LOXL1-AS1 silence on cell migration and invasion (Fig. 5E, F and Additional file 2: Fig. S2F-G). Overall, LOXL1-AS1 exacerbated HCC cell malignant behaviors via sponging miR-3614-5p to upregulate YY1 expression.

### YY1 transcriptionally activates LOXL1-AS1 in HCC cells

**Fig. 6:**
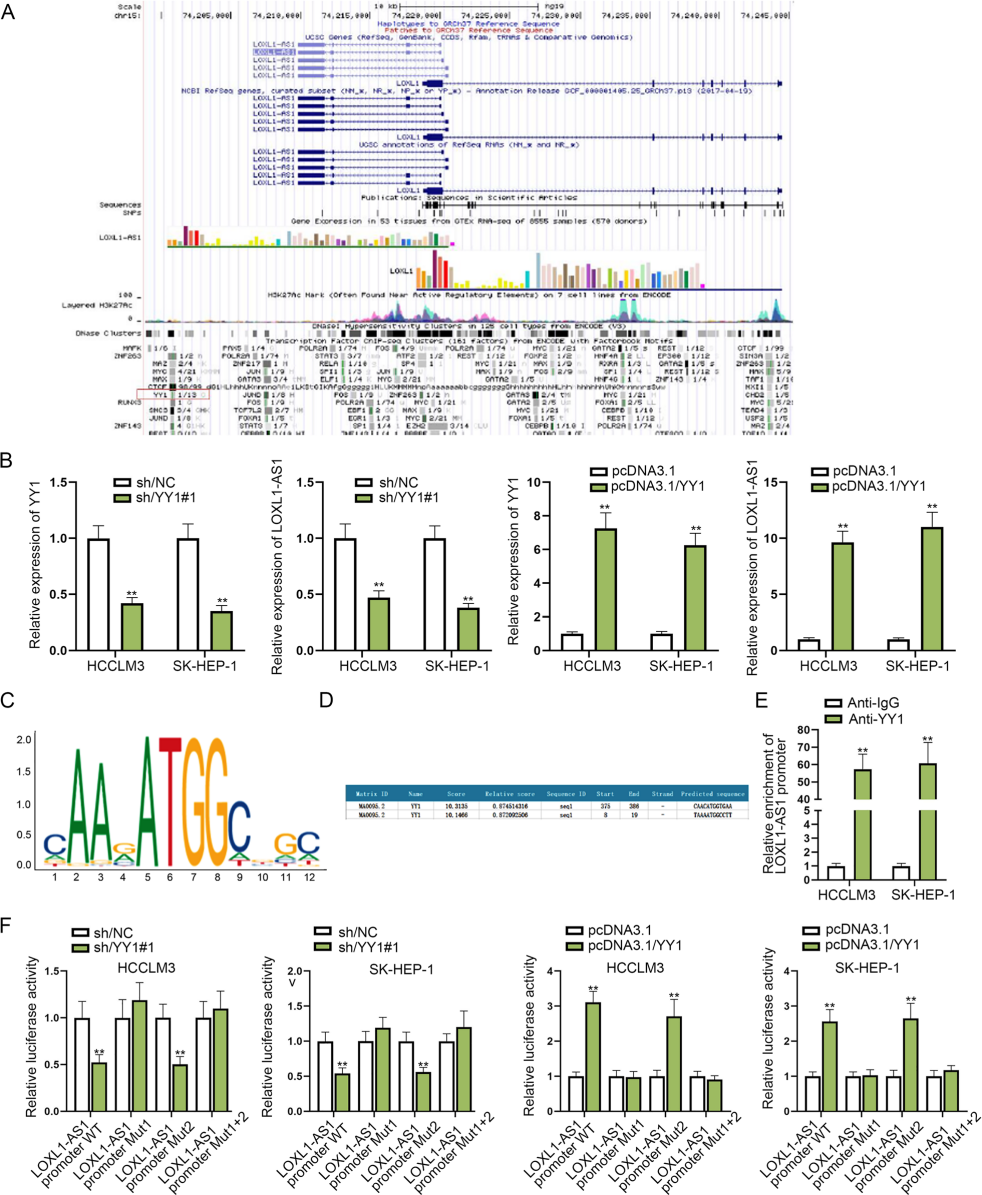
YY1 transcriptionally activates LOXL1-AS1 transcription in HCC cells. **A** UCSC database showed the possible transcription factors for LOXL1-AS1. **B** qRT-PCR analysis was done for the measurement of YY1 and LOXL1-AS1 levels in cells with transfection of sh/YY1 and pcDNA3.1/YY1. **C**, **D** DNA motif of YY1 and the binding sites of YY1 and LOXL1-AS1 promoter were obtained from JASPAR database. **E** ChIP assay proved the binding correlation between YY1 and LOXL1-AS1 promoter. **F** Luciferase reporter assay confirmed the binding of YY1 and LOXL1-AS1 promoter and detected the exact binding site. **P < 0.01

It has been indicated that transcription factors can form functional interactions with lncRNAs to transcriptionally activate or inactivate lncRNAs [26]. In order to explore the upregulation mechanism of LOXL1-AS1 in HCC cells, we searched for the upstream transcription factors of LOXL1-AS1. Through UCSC (http://genome.ucsc.edu/) database, we found YY1 might regulate the transcription of LOXL1-AS1 (Fig. 6A). Then we separately silenced or overexpressed YY1 in cells and then detected the corresponding changes of LOXL1-AS1 expression. Through qRT-PCR, we discovered that LOXL1-AS1 expression was lessened in cells when YY1 was silenced, while it was elevated in cells when YY1 was upregulated (Fig. 6B). These results indicated YY1 could positively regulate the expression of LOXL1-AS1. Subsequently, the DNA motif of YY1 and the two binding site of YY1 on LOXL1-AS1 promoter were predicted on JASPAR (http://jaspar.genereg.net/) database (Fig. 6C, D). Furthermore, ChIP assay was performed to verify the combination of YY1 and LOXL1-AS1 promoter. As abundant enrichment of LOXL1-AS1 promoter in anti-YY1 group was observed, the binding between YY1 and LOXL1-AS1 promoter was confirmed (Fig. 6E). Finally, luciferase reporter assay demonstrated that the luciferase activity of wild type LOXL1-AS1 promoter or LOXL1-AS1 promoter Mut2 was obviously repressed by YY1 depletion but promoted by YY1 upregulation, while the luciferase activity of LOXL1-AS1 promoter Mut1/Mut1 + 2 remained hardly changed, indicating that YY1 bound to LOXL1-AS1 promoter on site 1 (Fig. 6F). Thus, we concluded that YY1 transcriptionally activated LOXL1-AS1. Importantly, YY1 and LOXL1-AS1 constructed a positive feedback loop in HCC cells.

## Discussion

Hepatocellular carcinoma (HCC) is one of the most common malignant tumors with high incidence and mortality. LncRNAs have been confirmed as crucial regulators in human cancers, including HCC [27]. In HCC, various lncRNAs have been identified to function as oncogenes or tumor suppressors, such as MIAT [28], HOXD-AS1 [29] and PDPK2P [30]. Previous research has supported that LOXL1-AS1 can promote the development of different cancers, including pancreatic cancer [15], gastric carcinoma [16], non-small cell lung cancer [17] and HCC [17]. In this research, we mainly investigated the specific function and molecular mechanism of LOXL1-AS1 in modulating HCC cell behaviors. First of all, the upregulation of LOXL1-AS1 in HCC tissues and cell lines was discovered by bioinformatics analysis and qRT-PCR. Then we silenced LOXL1-AS1 in HCC cells for conducting loss-of-functional assays. As we expected, LOXL1-AS1 depletion restrained cell proliferation, migration and invasion while enhancing cell apoptosis in HCC. Thus, we concluded that LOXL1-AS1 promoted the malignant behaviors of HCC cells.

A flow of research has indicated that competing endogenous RNA (ceRNA) hypothesis exerts the vital role on molecular biological mechanisms in cancer development [24]. Furthermore, it has been confirmed that lncRNAs can function as a ceRNA to sponge miRNAs, so as to regulate mRNAs level at post-transcriptional level [25]. For instance, SNHG5 has been found to promote nasopharyngeal carcinoma development via regulating miR-1179/HMGB3 axis [31]. LncRNA KCNQ1OT1 has been ascertained as a sponge of miR-4458, thus facilitating osteosarcoma cell growth via upregulating CCND2 [32]. Herein, we detected the ceRNA possibility of LOXL1-AS1 in HCC cells. After utilizing bioinformatics tools and subcellular fraction assays, we confirmed that LOXL1-AS1 was a cytoplasmic lncRNA and could regulate genes at post-transcriptional level. Accordingly, we searched for the candidate miRNAs for LOXL1-AS1 through bioinformatics analysis and conducted mechanism assays for further screening and validation. Then we discovered that LOXL1-AS1 could sponge miR-3614-5p in HCC cells. MiR-3614-5p displayed low expression in HCC tissues and cells and overexpression of miR-3614-5p totally reversed the inhibitory effect of LOXL1-AS1 knockdown on HCC cell malignant behaviors. It has been reported that miR-3614-5p could repress cell proliferation and invasion of non-small cell lung cancer [33], which further supported the anti-tumor role of miR-3614-5p in HCC cells.

Furthermore, we proved that YY1 was the target gene of miR-3614-5p and negatively regulated by miR-3614-5p. YY1 is a zinc finger transcription factor that belongs to the GLI-Krüppel gene family 8 and dysregulated in assorted human cancers. For example, YY1 can accelerate laryngeal tumorigenesis and progress via repressing MYCT1 [34]. Importantly, YY1 can also facilitate HCC cell proliferation and tumor growth [35, 36]. In our research, we discovered that YY1 was high expressed in HCC tissues and cells and accelerated cell proliferation, migration and invasion while impeding HCC cell apoptosis. Rescue assays also proved that overexpression of YY1 could reverse the inhibitory effect of silencing LOXL1-AS1 on HCC cell malignant phenotype. Moreover, as a transcription factor, YY1 can transcriptionally regulate the expression of lncRNAs in cancer cells. For example, it has been certified that LINC00673 activated by YY1 can facilitate breast cancer development by regulating Hippo signaling pathway [37]. Also, YY1-induced ZFPM2-AS1 has been discovered to promote cell growth in lung cancer by upregulating TRAF4 [38]. Similarly, in our research, we discovered that YY1 could positively modulate LOXL1-AS1 expression by binding with LOXL1-AS1 promoter. Thus, the positive feedback pathway of LOXL1-AS1 and YY1 was constructed in HCC cells. However, the correlation between LOXL1-AS1/miR-3614-5p/YY1 in HCC patients was not investigated in current study for lack of patient samples, which would be one of our main targets in the future research. Moreover, in addition to animal experiments, a larger-scale of clinical samples needs to be collected to further confirm the crucial effects of LOXL1-AS1/YY1 axis on HCC progression.

Overall, our study proved that LOXL1-AS1 was high expressed in HCC cells. LOXL1-AS1 sponged miR-3614-5p to upregulate YY1, thereby accelerating cell proliferation, migration and invasion, but inhibiting cell apoptosis in HCC. Importantly, YY1 transcriptionally activated LOXL1-AS1, thus forming the positive feedback pathway in HCC cells. These discoveries may provide a novel insight for understanding HHC, which might ultimately benefit the discovery of new, effective therapeutic targets of HCC in the future.

## Materials and methods

### Cell culture

Three HCC cell lines (HCCLM3, Huh-7 and SK-HEP-1) and one normal liver cell line (THLE-2) were utilized for this study. SK-HEP-1 and THLE-2 cells were purchased from American Type Culture Collection (ATCC; Manassas, VA, USA). HCCLM3 and Huh-7 were purchased from Japanese Collection of Research Bioresources (JCRB) Cell Bank. We cultured THLE-2 cells in BEGM medium (Gibco, Grand Island, NY, USA) supplemented with 10% FBS, 100 U/mL penicillin and 100 μg/mL streptomycin (Beyotime, Shanghai, China), and cultured HCCLM3, Huh-7 and SK-HEP-1 cells in DMEM medium (Gibco) with the same supplements. All cells were cultured in a humidified atmosphere containing 5% CO_2_ at 37 °C.

### Cell transfection

HCCLM3 and SK-HEP-1 cells were seeded in 6-well plates for performing plasmid transfection with Lipofectamine 3000 (Invitrogen). Specific shRNAs for LOXL1-AS1, YY1 and their negative control (NC) were obtained from GenePharma (Shanghai, China). In addition, miR-3614-5p inhibitor/mimics and NCs were synthesized by Ribobio (Guangzhou, China). Overexpression plasmid of YY1 was obtained by inserting full-length of YY1 cDNA sequence into pcDNA3.1 vector, and the empty vector served as NC.

### qRT-PCR analysis

TRIzol Reagent (Invitrogen, Carlsbad CA, USA) was applied for extracting the total RNA in line with the protocols of supplier. Subsequently, the extracted RNA was subjected to reverse transcription into cDNA with PrimeScript Reverse Transcriptase Kit (Takara, Shiga, Japan) for conducting qPCR by SYBR Green PCR Kit (Takara). Finally, the relative expressions of genes were counted with 2^−ΔΔCt^ method. U6 or GAPDH served as internal reference.

### Colony formation assay

Transfected HCCLM3 and SK-HEP-1 cells were put in 6-well plates at the density of 600/well. After 14 days, colonies were fixed in 4% paraformaldehyde and stained by 0.5% crystal violet solution. In the end, colonies were imaged and counted manually.

### EdU assay

The proliferationHCCLM3 and SK-HEP-1 were determined with BeyoClick™ EdU Cell Proliferation Kit (Beyotime, Shanghai, China). After transfection, cells (1 × 10^4^) were plated into 96-well plates and treated for 2 h with EdU kit. DAPI solution was utilized to stain cell nuclei. Finally, EdU positive cells were observed by Olympus fluorescence microscope (Olympus, Tokyo, Japan) and calculated to reflect cell proliferation.

### TUNEL assay

The transfected HCCLM3 and SK-HEP-1 cells (1 × 10^4^) were washed in PBS, and then fixed with 4% PFA. Based on the user guide, TUNEL reagent (Merck KGaA, Darmstadt, Germany) was utilized to stain the apoptotic cells, and cell nuclei were counterstained with DAPI solution. Finally, we observed the positive cells via the fluorescence microscopy (Olympus).

### Flow cytometry assay

In accordance with the user protocols, cell apoptotic capability was evaluated via Annexin V-FITC apoptosis kit. Simply put, transfected cells were collected and rinsed by PBS for two times, followed by 10-min staining with Annexin V-FITC in dark room. Flow cytometer was applied for observation.

### JC-1 assay

Cell apoptosis was estimated through the change in mitochondrial membrane potential (Δψm) utilizing JC-1 assay. After transfection, 1 mL of cell suspension in 6-well plates were cultivated for half an hour with 2.5 µg/ml of JC-1 dye at room temperature, and then observed through microscope (Olympus).

### Transwell assay

After transfection, 1 × 10^4^ HCCLM3 and SK-HEP-1 cells in serum-free medium were seeded into the upper chamber of transwell inserts (Coring, Coring, NY, USA) with or without 30 μg of Matrigel (BD Biosciences, Franklin Lakes, NJ, USA), and the complete culture medium with 20% FBS was added into lower chamber. After 24 h, cells on the bottom of membrane were fixed and then stained by crystal violet. Finally, optical microscopy (Olympus) was applied for observation.

### Subcellular fractionation

HCCLM3 and SK-HEP-1 were lysed in cell fractionation buffer, and then were subjected to centrifugation for the supernatant. The nuclear pallet was lysed in cell disruption buffer and centrifuged. qRT-PCR was applied for detecting the LOXL1-AS1 content in cytoplasm and nucleus.

### FISH assay

The specifically designed RNA-FISH probe for LOXL1-AS1 was obtained from Ribobio and utilized in line with user guides. The air-dried cells were prepared for culturing with the LOXL1-AS1 FISH probe in hybridization buffer. Hoechst solution was applied for staining cell nuclei. In the end, we utilized the fluorescence microscopy for observation.

### RIP assay

RIP assay was conducted with the aid of Magna RIP™ RNA-Binding Protein Immunoprecipitation Kit (Millipore, Bedford, MA, USA). After the treatment with RIP lysis buffer, the cell lysates were harvested and cultured with anti-Ago2 or anti-IgG conjugated with magnetic beads. Then, the precipitated RNAs were retrieved and analyzed by qRT-PCR.

### RNA pull down

Based on the user guides, this assay was performed with the Pierce Magnetic RNA–Protein Pull-Down Kit (Thermo Fisher Scientific, Waltham, MA, USA). The gathered RNA extracts from cells were prepared to mix with the biotin-labeled probes of wild-type miR-3614-5p (Bio-miR-3614-5p-WT), biotinlyated mutant miR-3614-5p probe (Bio-miR-3614-5p-Mut) or NC probe (Bio-NC), with addition of magnetic beads for 1 h. The purified RNA levels were detected through qRT-PCR.

### ChIP assay

After nuclear extracts added with 1% formaldehyde, cross-linked chromatin was collected. The cross-linked chromatin was lysed and DNA in the length of 300 to 1000 base pairs was sheared by sonication. Then, 50% slurry of salmon sperm DNA/Protein A Agarose was applied for incubating the pre-cleared lysates. Next, the lysates was cultivated with anti-YY1 or anti-IgG. In the end, PCR was applied for analysis after extracting the precipitated DNA fragments.

### Luciferase reporter assay

Cells were subjected to co-transfection with the pmirGLO vectors (Promega, Madison, WI, USA) containing the wild-type (WT) and mutant (Mut) miR-3614-5p binding sites to LOXL1-AS1 fragment or YY1 fragment as well as miR-3614-5p mimics or NC mimics for 48 h. Luciferase Reporter Assay System (Promega) was utilized to estimate the relative luciferase activity (Promega). Further, LOXL1-AS1 promoter with wild-type or mutated YY1 binding sites were sub-cloned to the luciferase reporter pGL3 (Promega), then they were subjected to co-transfection with pcDNA3.1-YY1 or pcDNA3.1-NC.

### Western blot

The cells were lysed in cell lysis buffer. Proteins were extracted by sigma PROTTOT-1KT. Then, protein concentrations were measured using Bradford Protein Assay Kit. Later, SDS-PAGE was applied to isolate protein, and the protein was transferred to PVDF membrane, which was then co-cultured with specific primary antibodies, including anti-GAPDH and anti-YY1. Secondary antibodies were later added to incubate the membranes for 1 h at 37 °C. In the end, the protein expression was measured by chemiluminescence detection system.

### Statistical analysis

Each experiment was performed at least three times in this research. Mean ± standard deviation (SD) was used to show the results. Statistical analysis was achieved via SPSS 23.0. The Student’s *t* test, one-way analysis of variance (ANOVA) or two-way ANOVA was used for difference analysis. *P* < 0.05 was considered to indicate statistically significance in differences.

## Supplementary Information

**Additional file 1**. **Figure S1** (A) LOXL1-AS1 expression pattern in LIHC tissues and normal tissues was obtained from ENCORI. (B) Flow cytometry analysis was performed for the detection of apoptosis rate of sh/LOXL1-AS1 transfected cells. (C) Bioinformatics tool (ENCORI) was applied for predicting miR-3614-5p expression in both LIHC tissues and normal tissues. (D) YY1 expression in LIHC tissues and normal tissues was acquired on ENCORI. (E) YY1 protein level in different cell lines was measured via western blot. (F) YY1 protein level in HCCLM3 and SK-HEP-1 with or without sh/YY1 transfection was measured via western blot. (G) Cell apoptosis was analyzed via flow cytometry in response to sh/YY1 transfection. (H) YY1 protein level in different transfected groups was measured via western blot. **P < 0.01

**Additional file 2**. **Figure S2** (A–D) Representative images of Fig. 5A-D for colony formation, EdU, JC-1 and TUNEL assays were displayed. (E) Flow cytometry assay was conducted to evaluate cell apoptosis in response to transfection with indicated plasmids including sh/NC, sh/LOXL1-AS1#1, sh/LOXL1-AS1#1+inhibitor or sh/LOXL1-AS1#1+pcDNA3.1/YY1. (F-G) Representative images of Fig. 5E-F for transwell assay were demonstrated. **P < 0.01.

## Data Availability

Research data are not shared.

## Abbreviations

HCC: Hepatocellular carcinoma
LOXL1-AS1: Lysyl oxidase like 1 antisense RNA 1
YY1: Yin Yang 1
lncRNAs: Long noncoding RNAs
mRNAs: Messenger RNAs
miRNAs: MicroRNAs
ceRNA: Competing endogenous RNA
ATCC: American Type Culture Collection
JCRB: Japanese Collection of Research Bioresources
FBS: Fetal Bovine Serum
qRT-PCR: Quantitative real-time polymerase chain reaction
EdU: 5-Ethynyl-2'-deoxyuridine
DAPI: 4',6-Diamidino-2-phenylindole
Tunel: Terminal deoxynucleotidyl transferase (TdT) dUTP Nick-End Labeling
ChIP: Chromatin immunoprecipitation
FISH: Fluorescence in situ hybridization
RIP: RNA immunoprecipitation
WT: Wild-type
Mut: Mutant
SD: Standard deviation
ANOVA: Analysis of variance
