# Supplementary figures and images for: Study on the mechanism of LOXL1-AS1/miR-3614-5p/YY1 signal axis in the malignant phenotype regulation of hepatocellular carcinoma
Source: Biol Direct. 2021 Dec 4;16:24. doi: 10.1186/s13062-021-00312-8 (PMC8645132; doi:10.1186/s13062-021-00312-8)

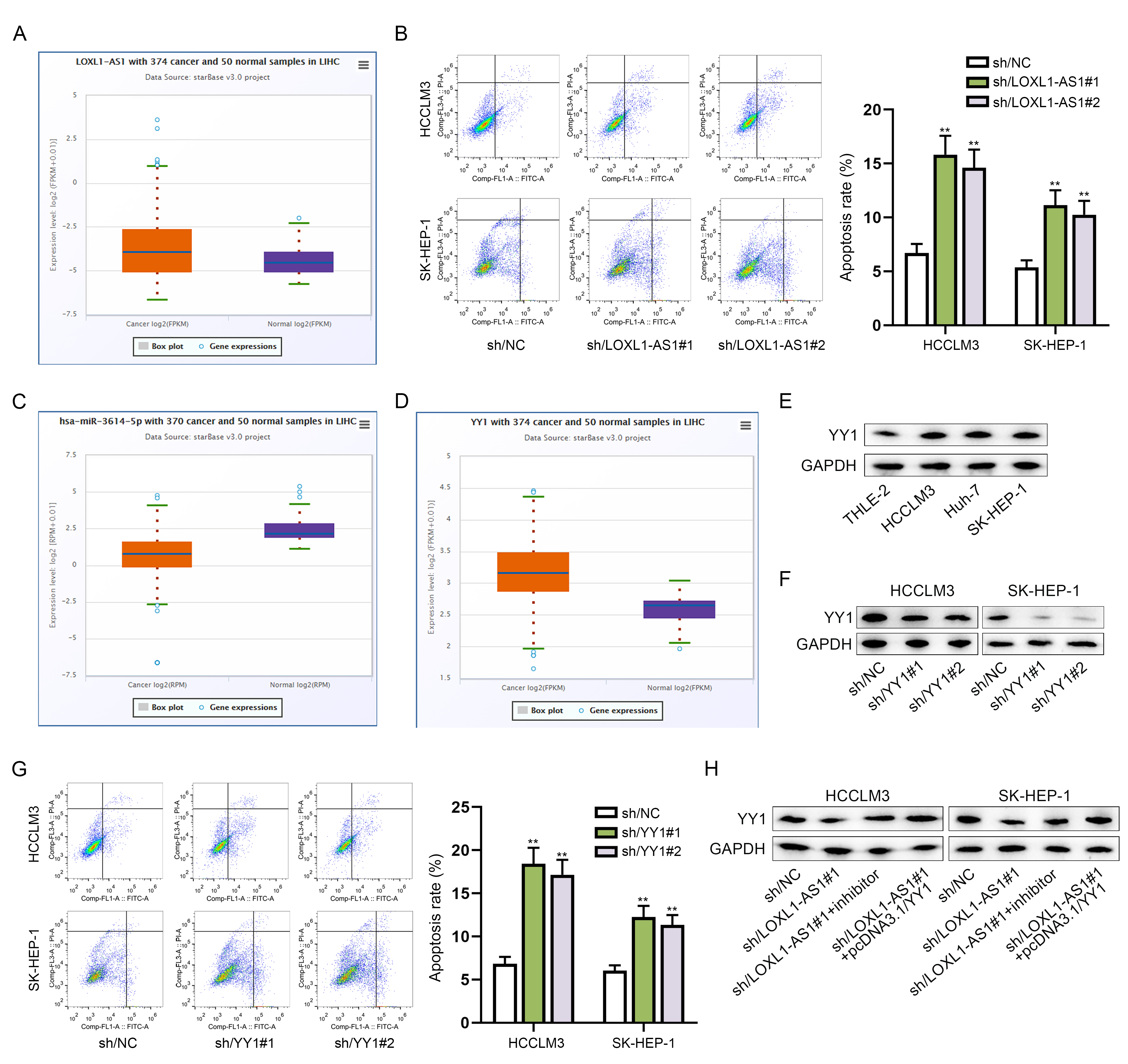

Supplement: Supplementary file 1 — Additional file 1. Figure S1 (A) LOXL1-AS1 expression pattern in LIHC tissues and normal tissues was obtained from ENCORI. (B) Flow cytometry analysis was performed for the detection of apoptosis rate of sh/LOXL1-AS1 transfected cells. (C) Bioinformatics tool (ENCORI) was applied for predicting miR-3614-5p expression in both LIHC tissues and normal tissues. (D) YY1 expression in LIHC tissues and normal tissues was acquired on ENCORI. (E) YY1 protein level in different cell lines was measured via western blot. (F) YY1 protein level in HCCLM3 and SK-HEP-1 with or without sh/YY1 transfection was measured via western blot. (G) Cell apoptosis was analyzed via flow cytometry in response to sh/YY1 transfection. (H) YY1 protein level in different transfected groups was measured via western blot. **P < 0.01 [file 13062_2021_312_MOESM1_ESM.tif]

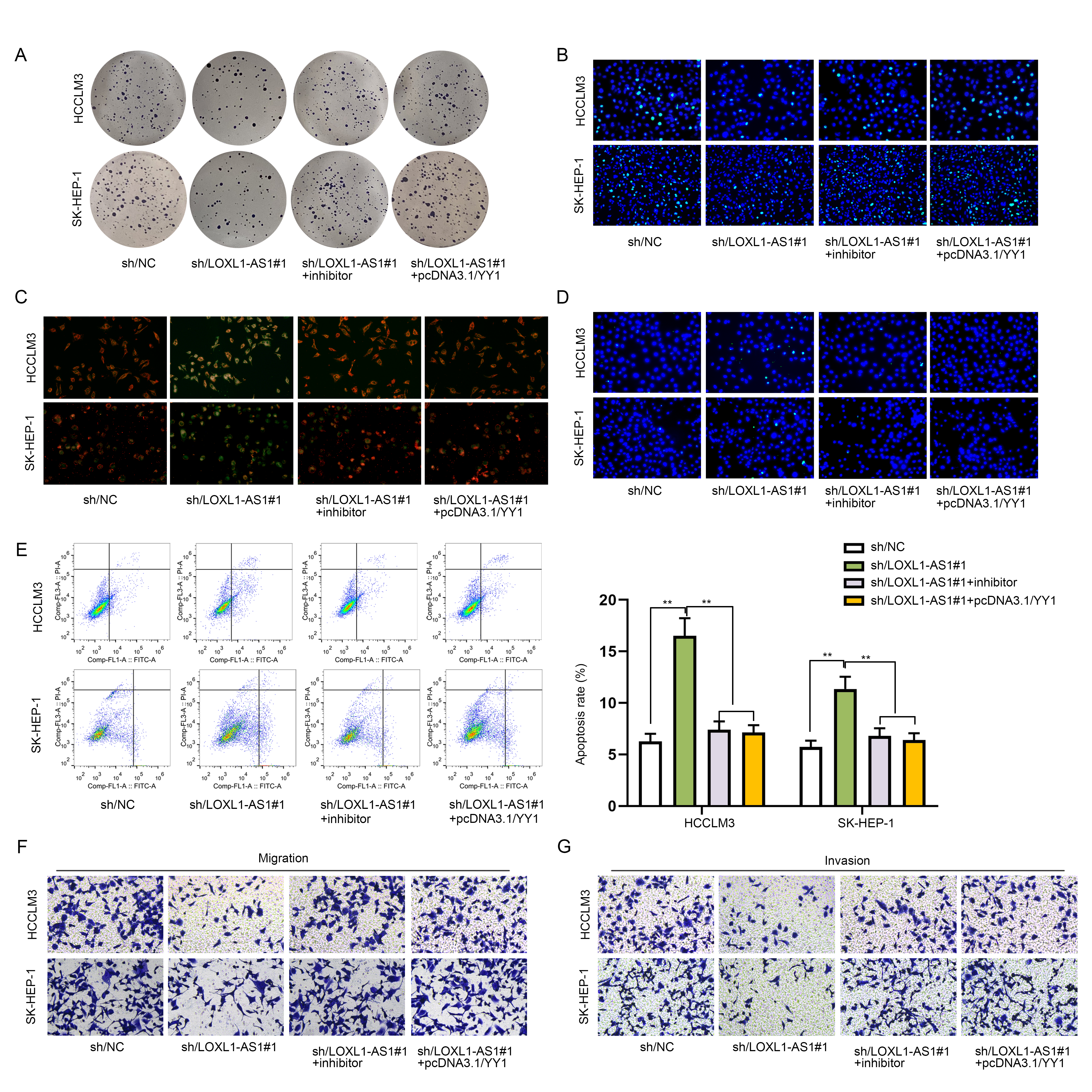

Supplement: Supplementary file 2 — Additional file 2. Figure S2 (A–D) Representative images of Fig. 5A-D for colony formation, EdU, JC-1 and TUNEL assays were displayed. (E) Flow cytometry assay was conducted to evaluate cell apoptosis in response to transfection with indicated plasmids including sh/NC, sh/LOXL1-AS1#1, sh/LOXL1-AS1#1+inhibitor or sh/LOXL1-AS1#1+pcDNA3.1/YY1. (F-G) Representative images of Fig. 5E-F for transwell assay were demonstrated. **P < 0.01. [file 13062_2021_312_MOESM2_ESM.tif]
